# Detection of epileptic spasms using foundational AI and smartphone videos

**DOI:** 10.1038/s41746-025-01773-1

**Published:** 2025-06-17

**Authors:** Gadi Miron, Mustafa Halimeh, Simon Tietze, Martin Holtkamp, Christian Meisel

**Affiliations:** 1 Computational Neurology, Department of Neurology, Charité—Universitätsmedizin Berlin, Berlin, Germany; 2 Berlin Institute of Health, Berlin, Germany; 3 Epilepsy-Center Berlin-Brandenburg, Institute for Diagnostics of Epilepsy, Berlin, Germany; 4 Epilepsy-Center Berlin-Brandenburg, Department of Neurology, Charité—Universitätsmedizin Berlin, Berlin, Germany; 5 NeuroCure Cluster of Excellence, Charité—Universitätsmedizin Berlin, Berlin, Germany; 6 Bernstein Center for Computational Neuroscience, Berlin, Germany; 7 Center for Stroke Research Berlin, Berlin, Germany

**Keywords:** Epilepsy, Paediatric neurological disorders

## Abstract

Infantile epileptic spasm syndrome (IESS) is a severe neurological disorder characterized by epileptic spasms (ES). Timely diagnosis is crucial, but it is often delayed due to symptom misidentification. Smartphone videos can aid in diagnosis, but the availability of specialist review is limited. We fine-tuned a foundational video model for ES detection using social media videos, thus addressing this clinical need and the challenge of data scarcity in rare disorders. Our model, trained on 141 children with 991 ES and 127 children without seizures, achieved high performance (area under the receiver–operating-characteristic curve (AUC) 0.96, 82% sensitivity, 90% specificity) including validation on external datasets from social media derived smartphone videos (93 children, 70 seizures, AUC 0.98, false alarm rate (FAR) 0.75%) and gold-standard video-EEG (22 children, 45 seizures, AUC 0.98, FAR 3.4%). We demonstrate the potential of smartphone videos for AI-powered analysis as the basis for accelerated IESS diagnosis and a novel strategy for the diagnosis of rare disorders.

## Introduction

Timely diagnosis of rare neurological disorders remains a significant challenge in healthcare, often resulting in delayed patient treatment^1^. Infantile epileptic spasm syndrome (IESS), a developmental and epileptic encephalopathy affecting approximately 1 in 2000–2500 infants in their first year of life, exemplifies this problem^2,3^. While approximately 9% of children experience some sort of paroxysmal movement events during their first year of life (most of which are benign), a smaller percentage actually have seizures^4^. Despite the stereotypical nature of the hallmark seizures of IESS, epileptic spasms (ES), diagnosis is frequently delayed by weeks to months due to misidentification of symptoms as benign physiological occurrences or failure to recognize any abnormality by physicians or parents^5–12^. These delays are associated with long-term poor cognitive outcomes, inadequate seizure control, increased disability, and higher healthcare costs^7,13^.

The widespread availability of smartphones and advancements in artificial intelligence (AI) may open new avenues for digital health technologies and accelerated neurological diagnostics. In epilepsy, videos captured by smartphones have already been shown to enhance diagnostic accuracy and clinical decision-making while reducing patient and family stress^14^. In the hands of experts, these videos facilitate earlier arrival to clinics, faster diagnostic EEGs, and improved treatment responses for children with IESS^15^. Videos from smartphones have furthermore been shown to be at times non-inferior to gold-standard video-EEG monitoring for initial diagnosis, offering advantages particularly in resource-limited settings^16,17^. Thus, while smartphone videos could in principle enable more rapid and accurate identification of ES, the shortage of medical professionals available for timely review and evaluation of patient videos limits the broader applicability of this approach. Rapid video evaluations supported by AI may help to broadly scale expert knowledge in detecting paroxysmal movements suspicious for seizures in infants and young children as the basis for faster diagnosis and treatment. Developing accurate AI models for rare conditions like IESS, however, presents unique challenges, primarily due to the scarcity of large, labeled datasets required for effective model training.

**Fig. 1:**
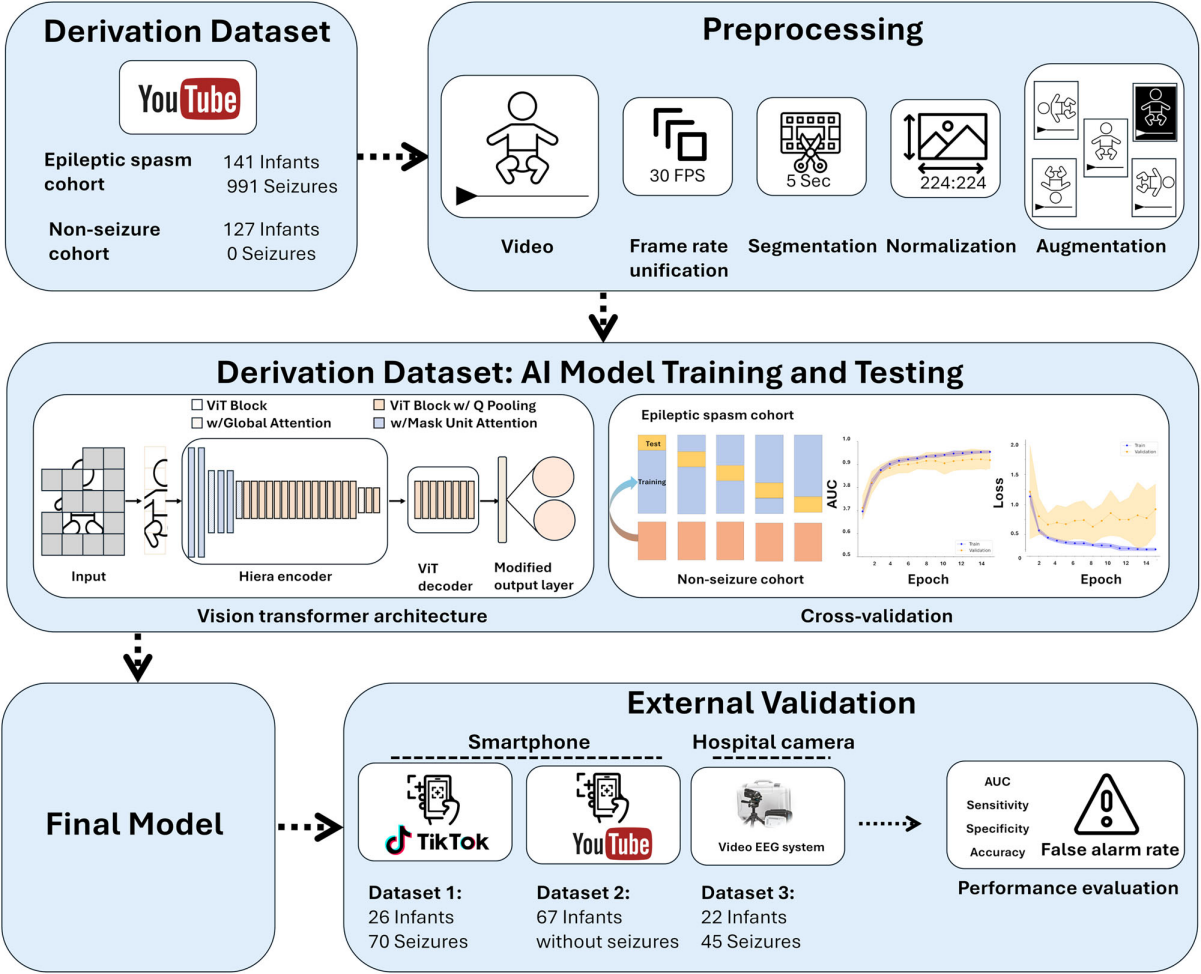
Study design. To acquire the derivation dataset, we conducted a search for YouTube videos with the keywords “epileptic spasms”, “infantile spasms”, and “West syndrome”. All video segments were reviewed by expert neurologists and underwent preprocessing. Videos were analyzed by a vision transformer model that was re-trained for the purpose of epileptic spasm identification. A five-fold cross-validation approach was used for training and testing. Finally, one single model was derived from the entire derivation dataset and tested on three external validation datasets: two datasets sourced from smartphone videos, and one dataset from gold-standard video-EEG monitoring. Performance on these validation datasets was assessed using the area under the receiver–operating-characteristic curve (AUC), sensitivity, specificity, accuracy, and false alarm rate.

Here, we address the challenge of timely IESS diagnosis with smartphone-based detection of ES by leveraging two key developments: powerful foundation vision models pretrained on extensive datasets and the utilization of the wealth of publicly available video data in social media. First, foundation vision models based on transformer architectures, trained on extensive datasets of images and videos from the internet, have been robust for human activity recognition^18,19^. These models have the potential to significantly enhance video-based seizure detection, extending capabilities to additional video sources, including smartphone recordings. This is particularly valuable given the variability in video quality, recording equipment, and patient demographics encountered in real-world settings. Second, social media platforms have inadvertently become valuable repositories of medical information, with user-uploaded videos clearly demonstrating the semiology of stereotypical seizures. While medical research has begun to leverage social media data for various purposes, this approach remains largely unexplored for rare neurological disorders and epilepsy. This untapped resource offers a potential solution to the data scarcity problem, enabling the development of robust AI models for conditions that traditionally lack sufficient clinical data. We curated a large dataset of ES seizures from open-source videos, thus addressing the data scarcity issue. We then trained an AI model for automated detection of ES, building on a state-of-the-art vision foundational model, thus benefiting from comprehensive pretraining. Finally, we validated our model on three additional independent datasets, comprising both smartphone recordings and gold-standard in-hospital video-EEG monitoring data, thus ensuring model robustness across varied video sources and clinical settings (Fig. 1).

## Results

### Derivation dataset: population characteristics, training, performance

For model training and testing, we collected social media smartphone videos from 141 children, comprising 991 ES and 597 non-seizure video segments of 5-s duration each (Fig. 1). The median number of segments per child was 6 (IQR 4–12, range 1–42). We further enriched the training dataset with 127 healthy infants, contributing 1385 video segments (median 8 per child, IQR 4–14, range 1–70; Table 1). Videos containing ES were published between 2007 and 2022, with a median of 8885 views per video (IQR 1687–42,274.5), 31 likes (IQR 8–121.5) and 5 comments (IQR 1–13.5). As videos were derived from social media, they exhibited considerable technical heterogeneity in terms of resolution, bitrate, brightness of the video, and sharpness (Table 1). Videos also exhibited semiological heterogeneity, with 60.9% having flexor, 18.7% extensor, 20.4% mixed semiology, and 23.4% of subjects having subtle spasms, characterized by slight movement involving face, eye or limbs (Supplementary Table 1).

**Table 1:** Study Population and Video Characteristics

	Derivation Dataset		External Validation Dataset		
	Epileptic Spasm Cohort	Normally Behaving Infants	Dataset 1—Smartphone—Epileptic Spasm Cohort	Dataset 2— Smartphone—Normally Behaving Infants	Dataset 3— Hospital- Video-EEG Monitoring
Number of subjects	141	127	26	67	22
Data source	YouTube	YouTube	TikTok, YouTube	YouTube	Video-EEG monitoring
Seizure segments	991	0	70	0	45
Non-Seizure segments	597	1385	31	666	11,190
Segments per child, median (IQR)	6 (4–12)	8 (4–14)	3 (2–5)	6 (2.75–10.5)	382 (158.25–766)
Resolution: high/medium/low%	57/7/36%	52/11/37%	88/8/4%	57/34/9%	2/77/21%
Bitrate, median (IQR) kbps	530 (301–1016)	949 (449–1637)	1113 (925–1403)	2146 (779–3243)	3538 (3404–3671)
Sharpness values, median (IQR), laplacian variance	88 (45–216)	44 (22–108)	303 (149–559)	60 (24–82)	284 (196–411)
Brightness, median (IQR) gray level intensity	104 (80–123)	112 (89–138)	117 (111–133)	124 (101–143)	107 (89–125)
Motion, median (IQR) frame absolute difference	175 (126–241)	182 (135–238)	192 (154–270)	171 (119–236)	77 (50–113)

High resolution is defined as above 720p, medium resolution between 480 and 720p, and low resolution under 480p. Abbreviations: *kbps* kilobytes per second, *IQR* interquartile (25th to 75th) range.

**Fig. 2:**
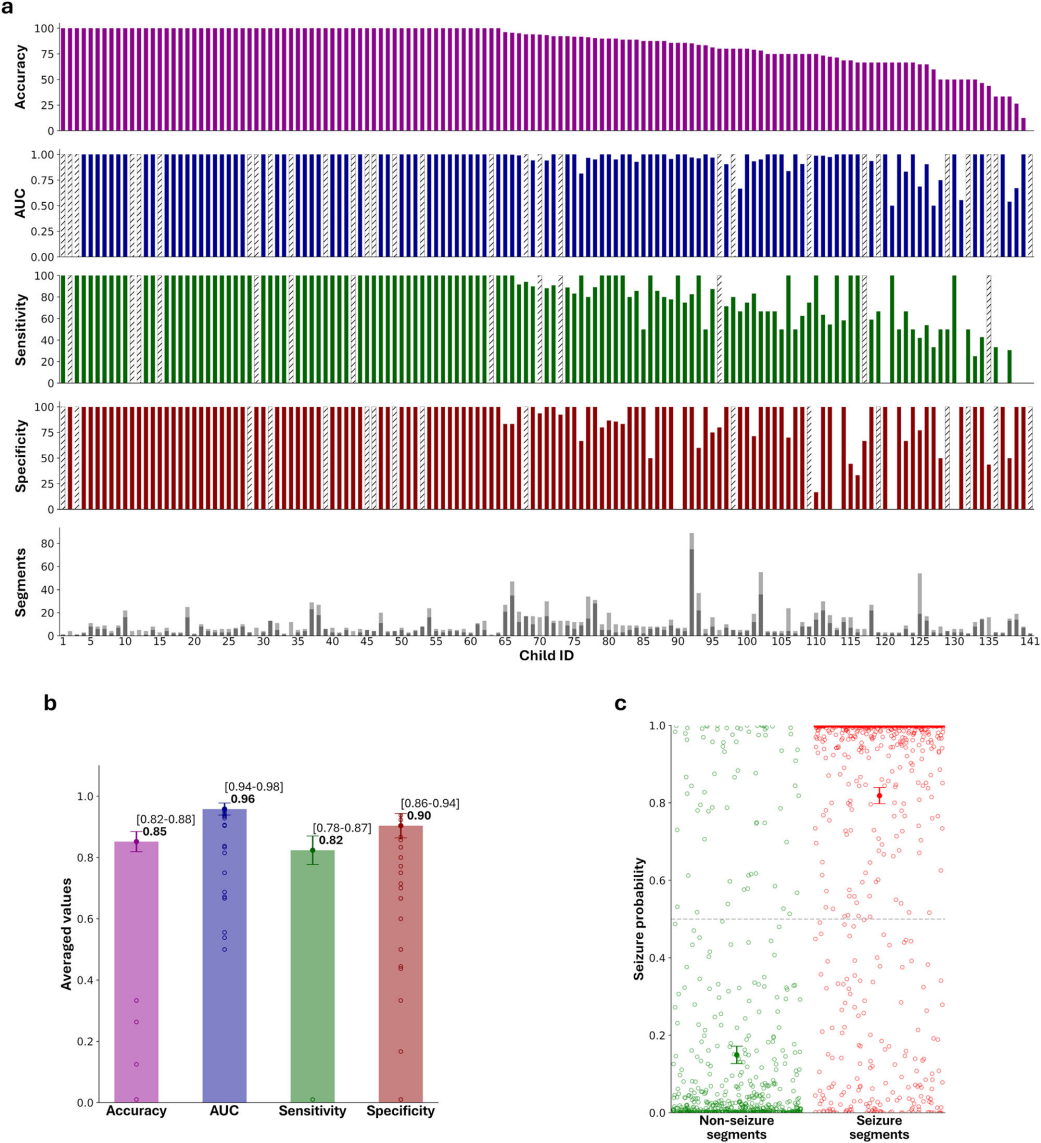
Seizure detection performance on the derivation dataset (smartphone videos). **a** Each column represents one subject. Only children who had both seizure and normal segments (111/141) were included in calculating AUC (in cases where only one class of videos was available, a gray column is depicted). **b** Overall metrics of performance across all subjects. **c** All model predictions, a threshold of 0.5 is depicted as a dashed line. Markers denote mean and 95% confidence intervals. Abbreviations: AUC area under the receiver-operating-characteristic curve.

Our model detected ES with an area under the receiver–operating-characteristic curve (AUC) of 0.96 (CI 0.94–0.98, five-fold cross-validation) on the derivation dataset. Using a threshold of 0.5, this provided a sensitivity of 82% (CI 78–87%), specificity of 90% (CI 86–94%), and accuracy of 85% (CI 82–88%) on the test folds (Fig. 2). No statistically significant relationships were found between the technical characteristics of videos and prediction performance in the derivation dataset (Supplementary Table 2).

### External validation: population characteristics, performance, false alarm rate

**Fig. 3:**
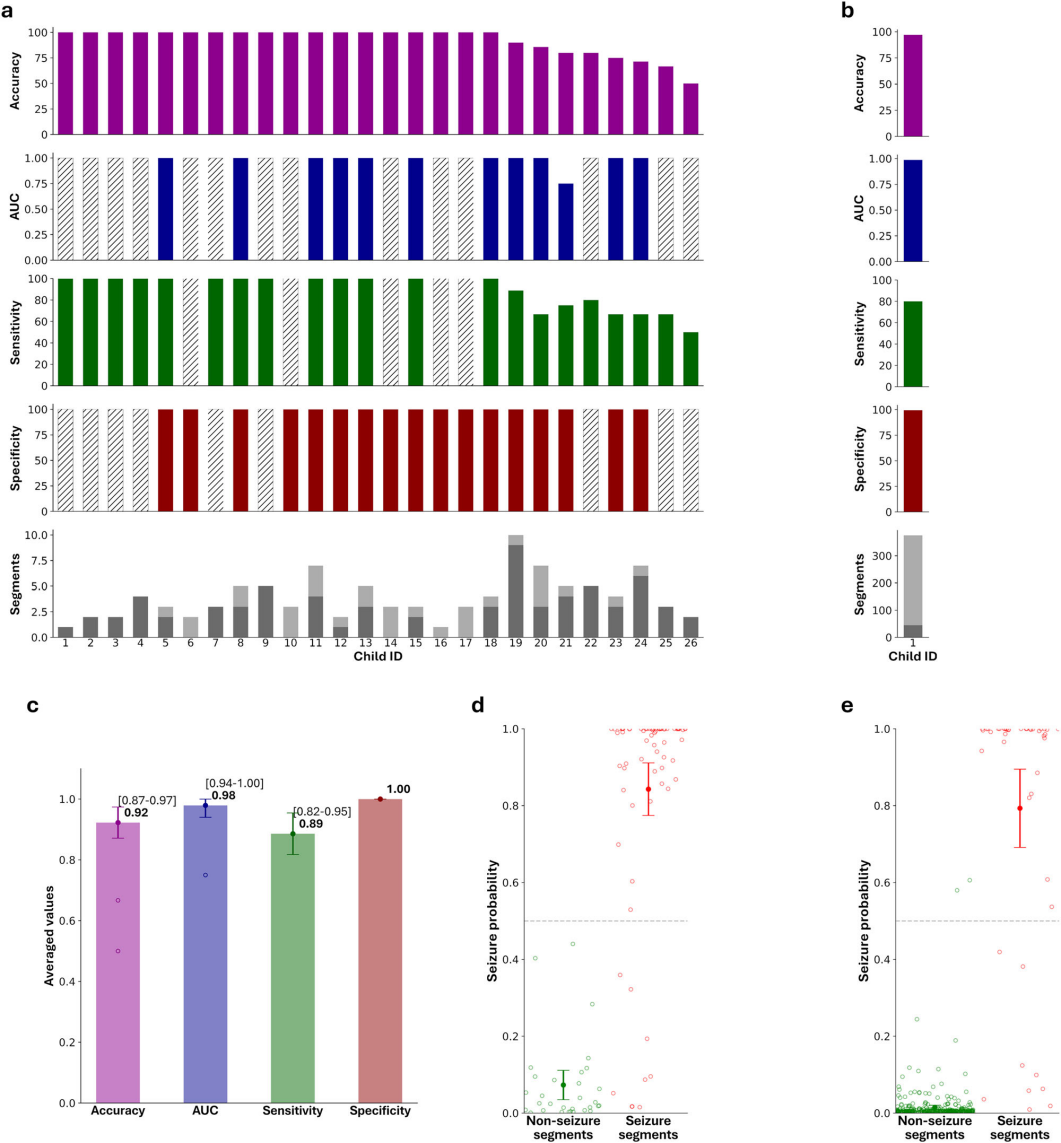
Performance of classification model on external validation datasets (smartphone and gold-standard video-EEG). **a**, **b** Individual performance metrics for 26 children with epileptic spasms included in the external validation dataset 1 (panel **a**, smartphone-derived) and for one child with epileptic spasms in external validation dataset 3 (panel **b**, hospital-derived). Each column represents one subject. Only children who had both seizure and normal segments were included in calculating AUC (in cases where only one class of videos was available, a gray column is depicted). **c** Overall metrics of performance across all subjects. **d**, **e** All model predictions are shown. Markers denote mean and 95% confidence intervals. Abbreviations: AUC area under the receiver–operating-characteristic curve.

Using the fixed, trained algorithm with a fixed threshold, we next evaluated our seizure detection model on three independent datasets to assess out-of-sample performance, establish FAR, and evaluate transferability to different video sources. Dataset 1, smartphone-based, included 26 infants with ES (70 seizure and 31 non-seizure 5-s video segments). Our model achieved high performance, comparable to the derivation dataset testing, with an AUC of 0.98 (95% CI: 0.94–1.0), sensitivity 89% (95% CI: 82–95%), specificity 100% (95% CI: 100–100%), and accuracy of 92% (95% CI: 87–97%; Fig. 3).

**Fig. 4:**
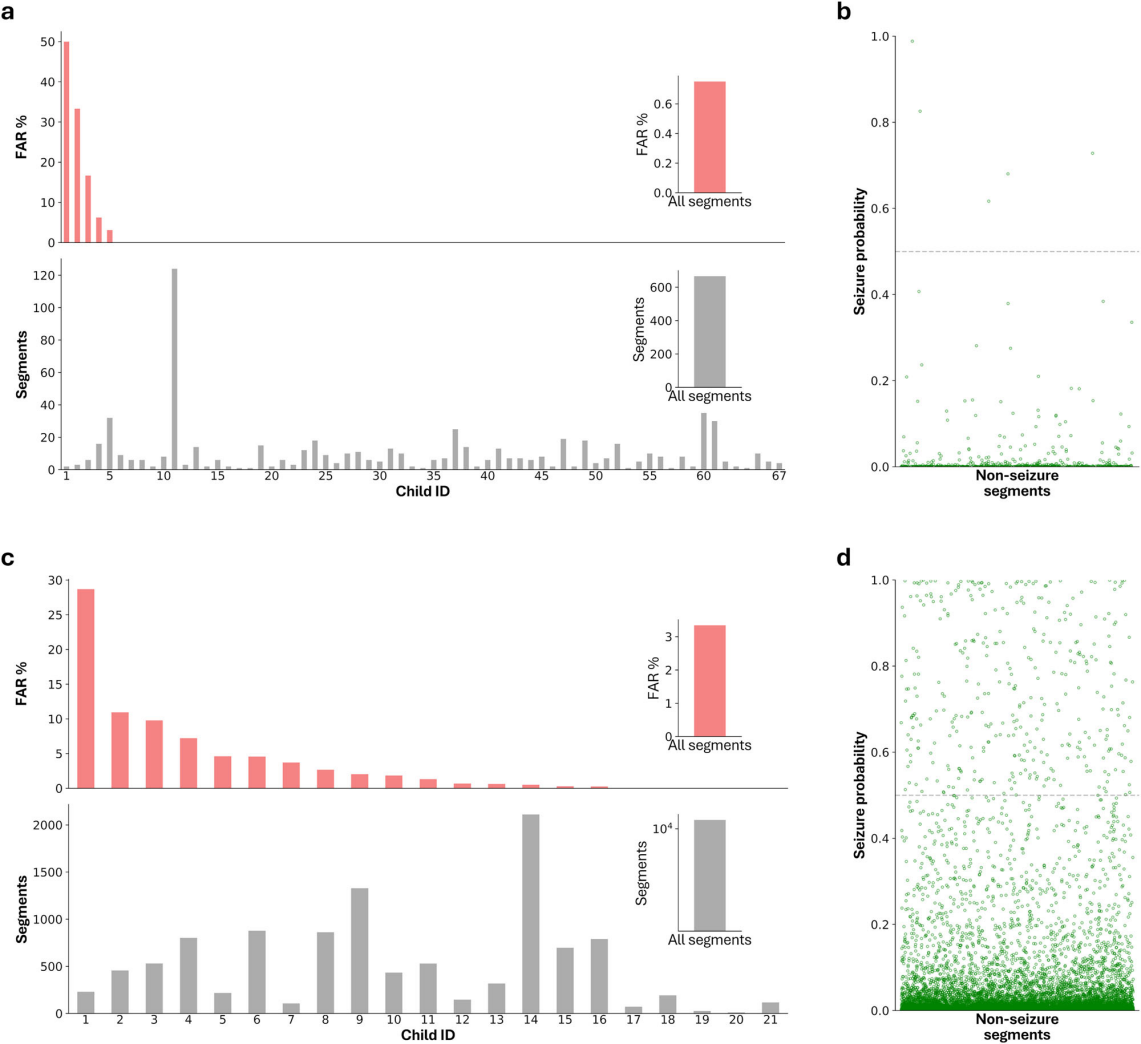
False alarm rate (FAR) evaluation. All subjects evaluated had no seizures; every positive prediction was considered a false alarm. The FAR is the percentage of positive predictions across all subjects. **a** FAR evaluation on an out-of-sample smartphone-derived video dataset including 67 children and 666 five-second segments (dataset 2). Each bar corresponds to one child. In total, 5 of 666 segments were classified as positive detections (0.75%), across all children, the average is 1.6%. **b** All the video segments are plotted with the prediction probability. **c** FAR evaluation on an out-of-sample gold-standard video EEG-derived dataset including 21 children and 10,860 five-second video segments (dataset 3). **d** All video segment predictions of the video-EEG dataset.

Dataset 2, also smartphone-based, included 67 normally behaving infants with 666 5-s segments. False detections were identified in 0.75% (5/666) of evaluated video segments, with 62 of 67 subjects having no false alarms. This resulted in a mean FAR per patient of 1.6% (95% CI: 0.0–3.4%; Fig. 4). Analysis of the 5 false positives revealed 3 cases with sudden bilateral arm extensions (prediction probabilities of 0.98, 0.82, 0.61), 1 with camera movement artifacts (probability: 0.68), and 1 with sudden limb flexion (0.72).

In both smartphone-based external validation datasets, no statistically significant relationships were found between the technical characteristics of videos and prediction performance (Supplementary Table 2). Furthermore, there were no statistically significant differences in semiology of seizures between ES subjects in the derivation and validation datasets (Supplementary Table 1).

Dataset 3 was sourced from in-hospital gold-standard video-EEG monitoring camera systems. In 21 infants without seizures, false detections occurred in 3.4% (365/10,860) of all video segments, with a mean FAR per patient of 3.8% (95% CI: 1.0–6.5%; Fig. 4). As an additional control we assessed videos from one child with ES (45 seizure and 330 non-seizure segments), resulting in an AUC of 0.98, sensitivity of 80%, specificity of 99%, and accuracy 97% (Fig. 3), closely matching the derivation and external validation smartphone ES datasets. To note, the age of the child (mean and standard deviation 1.18 ± 0.41 years) was not significantly correlated to model performance metrics.

Finally, we investigated potential contributing factors to false detections across datasets and within dataset 3. Comparing across datasets, we found that dataset 3, which had the highest FAR, had significantly lower video resolution than the other datasets (*p* = 0.006, Chi-square test), with only 2% of video segments having a resolution above 720p, and 21% of videos having low resolution under 480p. Dataset 3 also included night-time footage, which was not present in the other datasets. Excluding night-time videos lowered the total FAR in dataset 3–2.8% (283/9937 video segments). Dataset 3 contained long-term, continuous monitoring of children in hospital rooms and was prone to additional sources of bias and obstructions, including EEG caps, bed cribs, and family member interference. To systematically assess the overall visibility of the child within the video, we used an object detection model and compared model confidence measures between videos with and without false detections. This confidence measure acted as a surrogate marker for child visibility. False positive videos showed significantly lower confidence for infant detection (median confidence of 0.23 in correctly detected vs 0.16 in false positive, *p* < 0.001, Mann–Whitney *U* test). In examining technical characteristics affecting FAR, false positive videos had significantly lower image sharpness (median Laplacian variance 207 vs. 286 in correctly detected videos, *p* < 0.001, Mann–Whitney *U* test), significantly lower bitrate (median kilobytes per second of 3496 vs. 3545 in correctly detected videos, *p* = 0.006, Mann–Whitney *U* test), and significantly lower motion intensity (73.4 vs. 89.1 median frame absolute difference). Collectively, these results thus suggest that false alarm rate (FAR) and seizure detection performance hinge on video quality and child visibility, both of which can be addressed when collecting new data from smartphones or in-hospital video cameras.

## Discussion

Rare disorders are often difficult to diagnose and treat, as they can be paroxysmal in nature and diagnostic tests, as well as specialist clinical expertise, are often not widely available. Ninety percent of rare disorders in childhood have major neurological effects, and AI technology holds promise in accelerating diagnosis but requires large datasets. Early identification of seizures is critical for accurate and timely diagnosis of early-age-onset epilepsy, particularly for IESS, a rare neurological disorder often marked by delayed diagnosis and treatment^5,6,11,12^. With the increasing availability of smartphones and the integration of AI into daily life, automated detection of seizures from videos has the potential to address this clinical need. Here, we curated a large, heterogeneous dataset of seizure videos from social media to develop and test an AI model capable of detecting ES with high performance. We validated our model across multiple independent datasets, demonstrating strong performance with low FARs. Notably, our model, initially trained on smartphone videos, also showed robust adaptability when applied to gold-standard video-EEG monitoring recordings, though with an increase in FAR in the hospital setting.

AI has been successfully applied to automate multiple tasks in the medical domain, including analysis of diagnostic imaging and electronic health records^20,21^. In epilepsy, AI has accelerated and improved various aspects of care, including EEG analysis, neuroimaging interpretation, seizure detection and forecasting based on data from wearable medical devices, and recently, seizure detection from video-EEG monitoring recordings^22,23^. While advancements in medical AI have been propelled by the development of datasets from various sources such as electronic health records, imaging, and histology, obtaining datasets for rare neurological disorders remains a significant challenge due to the limited availability of data and regulatory constraints on data sharing^20^. Establishing video datasets for medical vision AI is furthermore challenged due to issues related to patient privacy. In epilepsy, open-source datasets of epileptic seizure footage are generally very small and limited to educational purposes. We addressed these challenges by demonstrating the feasibility of collecting a clinically relevant dataset of a rare neurological condition using openly available data from social media. This approach allowed us to identify 167 infants with over 1000 ES. In comparison, a single tertiary center serving a large population of three million people would, on average, see approximately 24 such children per year, thus a similar-sized dataset would be expected to take years to develop^24^. Furthermore, this approach established a diverse, heterogeneous cohort, potentially reducing the need for model retraining, improving accessibility across different populations and boosting clinical applicability, addressing a common barrier for AI integration in everyday clinical practice^20^.

Previous studies have begun to explore the use of social media in medical AI development, demonstrating its growing potential. One study used YouTube to create a dataset for analysis of open surgery techniques and to assess surgical skills. Another study created an image-based foundational model for pathology based on histological images uploaded to Twitter^25,26^. In neurology, YouTube videos have been used as adjunctive data sources for development or testing of AI models in assessment of Parkinson’s disease, abnormal gait, essential tremor, facial paralysis, and autism^27–31^. However, our approach is unique in that it adds to this body of literature by using social media to create a dataset for a rare epileptic disorder, including one of the largest datasets in the field of automated video analysis of seizures^22^.

Our ES detection model performed with high accuracy, achieving an AUC of 0.96, sensitivity of 82% and specificity of 90%, as evaluated using a five-fold cross-validation approach and similar levels of performance on the out-of-sample test datasets. To develop the AI model, we adapted an existing large-scale vision transformer model, which had originally been trained for general action recognition tasks on a massive dataset of videos from the internet^18,19^. Through this, we reduced the amount of data required for training, making it a feasible approach for developing a medical AI model with limited training data, such as IESS. Our strategy of adapting a generalist foundational model to medical AI aligns with emerging trends in the field^32^. To our knowledge and based on recent reviews of video-based seizure detection, ours is the first approach utilizing the vision transformer architecture in the domain of video-based seizure detection. Previous methods predominantly relied on skeleton landmark identification, frame-based approaches combined with neural networks, or optical flow methods^22,33^. The vision transformer architecture offers several advantages over these more traditional approaches, including superior ability to capture long-range dependencies in video sequences, enhanced robustness to variations in camera angle and lighting conditions, and improved performance on subtle motion patterns characteristic of ES^34,35^.

According to best practice in the field, we validated our model on three additional out-of-sample datasets. In two independent smartphone-derived cohorts, our model performed similarly to the derivation cohort, achieving an AUC of 0.98 on a cohort of infants with ES. Notably, testing on a dataset of normally behaving infants, we found a FAR of 0.75%, with 62 of 67 children having no false detections. This is a key finding with regard to potential clinical applicability, as false alarms are a barrier to clinical adaptation of new diagnostic technology. To note, dataset 2 contained mostly videos from TikTok, a different social media platform from the derivation dataset, introducing an additional source of variability that did not affect the model performance. Furthermore, the model’s consistent performance across diverse smartphone videos, regardless of technical characteristics such as brightness, lighting or sharpness, suggests robust generalizability that would be valuable in real-world clinical applications where video quality cannot always be controlled.

While our model was trained exclusively on smartphone data, we also tested it on a cohort of infants undergoing gold-standard video-EEG (dataset 3), to assess robustness and transferability to other camera sources. The ability of an AI model to perform well on different video sources is a measure of generalizability and could enable translation to different clinical settings. The hospital-based dataset differed from the derivation dataset considerably, as these were long-term videos captured from wide-view stationary cameras that were distant from the subject, compared to smartphone videos. Infants wore EEG caps, resolution was significantly lower, there were often additional people (family members, medical staff) in the frame, and videos were recorded both during the day and at night. Nevertheless, our model achieved a comparable sensitivity on 45 seizures from a patient with ES, and on an additional 21 patients - comprising over 10,860 video segments without seizures—the total FAR was 3.4%. This higher FAR appears driven by several factors: reduced infant identification in the frame and significantly lower image resolution and sharpness, due to the use of stationary cameras with a wide view and obstructing factors in the hospital environment (as measured by lower confidence scores from our object detection model), and variable lighting conditions. Lower model performance was also observed in low-motion intensity video segments, most likely due to nighttime video being characterized by paucity of movement. Thus, excluding nighttime footage improved FAR to 2.8%. Although this FAR rate is higher than that observed in the smartphone data, it shows promise for potential future utilization in this clinical setting. Testing in future prospective studies should control for these confounders, particularly footage during nighttime, and future work should focus on incorporating video data from multiple camera sources within seizure detection training data to further improve transferability to different sources of data.

The field of video analysis for seizures in epilepsy is a developing one, with a recent review having identified 34 studies assessing video-based seizure detection^22^. This review revealed large heterogeneity in methods used, dataset size, and performance measures reported. The largest study to date reported the use of a dedicated audio/visual system for seizure detection and included 104 patients and a total of 2767 seizures. This study included multiple seizure types and detailed the clinical decision-making outcome of the intervention, but did not report machine learning performance measures such as sensitivity and specificity, somewhat limiting interpretability^36^. A prior phase 3 study by the same group did report these metrics, however, their approach was semi-automated with involvement of a clinical neurophysiology specialist in the loop^37^. In early-onset epilepsy, a notable phase 2 study aimed at detecting clonic seizures examined 12 infants in the hospital setting, and achieved a maximal AUC of 0.79 in discriminating a total of 78 clonic seizures from random movements or other seizure types. Other studies focusing on pediatric seizure have shown promise for multiple seizure types including tonic-clonic seizures^23^, nocturnal motor seizures^38^, and neonatal seizures^39–43^. We contribute to this literature by reporting a model trained on smartphone data rather than from in-hospital cameras or specialized hardware, yielding high performance, and assessing seizure detection in the in-field setting. Our study includes a large number of participants and seizures, focuses on ES − a subtle seizure semiology − and adheres to the reporting and testing standards set forth by the International League Against Epilepsy for validation of AI technology for seizure detection devices^44^.

Future implementation of our seizure detection model into clinical practice requires integration into healthcare workflows where it can serve as both a screening and decision support tool. As a prerequisite, a secure digital infrastructure must be established to enable safe and ethical communication between parents and providers. A smartphone application implementing our model could allow parents to record suspicious movements for rapid automated assessment, potentially expediting specialist referrals when warranted. For healthcare providers, the model could be deployed either through compliant cloud services or as locally installed software integrated with electronic health records, serving as decision support during initial evaluations and for monitoring treatment response. For example, primary care physicians could upload videos for automated analysis, helping identify seizures that might otherwise be missed, or neurologists could use the technology to quantify treatment response in already diagnosed patients. The potential benefits extend across multiple levels: for patients, earlier diagnosis and treatment initiation has been associated with improved seizure control, better cognitive development, and reduced disability^5–7,10–13^; for healthcare systems, streamlined diagnosis could reduce unnecessary visits, decrease emergency utilization, and lower long-term care costs associated with developmental delays. However, it must be emphasized that video analysis alone is not sufficient for definitive ES diagnosis, as certain non-epileptic paroxysmal events and seizure mimics (such as gastroesophageal reflux, benign sleep myoclonus, or exaggerated startle responses) cannot be reliably differentiated by visual assessment alone. Thus, our approach should be viewed as a tool to accelerate the pathway to gold-standard video-EEG diagnostic evaluation and appropriate medical treatment, rather than as a standalone diagnostic replacement. Future implementation research should focus on further validation in real-world settings, quantifying improvements in time-to-diagnosis, treatment initiation speed, and long-term patient outcomes.

This study has several limitations. First, in the derivation dataset, there was no gold-standard electrophysiologic or clinical data to confirm the presence of ES. However, videos were reviewed by two expert neurologists trained to identify ES semiology, and cases of subtle spasms where there were disagreements in the initial interpretation were resolved through discussion and consensus. Likewise, although unlikely, non-motor seizures could not be excluded from interictal videos due to the lack of EEG. However, these would not be expected to be identified through visual inspection by an expert as well, and external validation of the model was done on gold-standard confirmed infants with and without epilepsy. Second, since we did not have demographic or clinical data available for social media videos, it was not possible to fully exclude potential biases related to age, sex, or ethnicity. Furthermore, some participants had more video segments than others, and in order to maximize the dataset, we could not balance seizures to interictal segments in a 1:1 ratio. To address this limitation, data collected was highly heterogeneous, a large dataset was examined, and additional validation was conducted on completely independent datasets of normally behaving infants and infants with gold-standard video-EEG. Third, while ES are the predominant seizures in children with IESS, they may have additional seizure types, and expansion of our approach to additional seizures is necessary to have true clinical applicability. Furthermore, certain ES mimics may be underrepresented in the non-ES cohort and should be further evaluated in future studies. Fourth, the study design is limited by an inherent selection bias toward videos that were uploaded to social media, and future prospective studies are needed to further establish generalizability.

Our findings demonstrate a novel approach for AI model development suitable for rare neurological disorders. We address the clinical need for early detection of ES in infants by developing a high-performing video-AI model capable of identifying this seizure semiology from widely available smartphone videos. The model’s robust performance across multiple datasets, including gold-standard video-EEG recordings, underscores its potential for clinical application. While challenges remain, including further prospective validation in different clinical settings, this work lays a foundation for future developments in video-based automated seizure detection.

## Methods

### Derivation dataset collection and annotation

The study design, data collection, preprocessing, AI model development and testing are depicted in Fig. 1. We conducted a systematic search on YouTube videos published before 2022 using the keywords “infantile spasms”, “epileptic spasms”, and “West syndrome”. Videos were included based on the following criteria: (1) subject appears to be under 2 years of age, (2) video contains an event consistent with ES semiology, as independently confirmed by two expert neurologists (G.M. and C.M.). In cases where there was initial disagreement, both reviewers jointly re-reviewed the videos and reached consensus through discussion, (3) subject is clearly visible and not substantially obstructed by other people, objects or overlying text, (4) video quality (resolution, lighting) is sufficient for visual recognition of semiology, without obscuring filters or effects. Exclusion criteria were: (1) subject or limbs obstructed from view (e.g., close-ups of the face or trunk), (2) insufficient video quality for semiology determination, (3) subject is clearly older than 2 years.

Sample sizes and case-control ratios were not pre-determined in order to maximize data inclusion. To ensure a diverse population, videos were included regardless of the country of origin, language spoken, video length, or upload year. ES were included regardless of the exact semiology, thus ensuring inclusion of as wide a variety of ES symptomatology as possible, including subtle spasms as characterized in prior literature^45–47^. Next, non-overlapping 5-s segments were manually reviewed and annotated, either containing stereotypical movements for ES or non-seizure segments. ES were randomly positioned within the 5-s windows, and in cases of seizure clusters, multiple seizures within a segment were allowed.

To further enhance our training dataset, we incorporated additional videos of normally behaving infants from previously collected YouTube datasets, reflecting a large variety of infant activities^48–50^. By doing so, we specifically aimed to include a diverse range of movements. For control videos, we applied parallel inclusion criteria: (1) subject appears to be under 2 years of age, (2) subject is clearly visible, active, and not substantially obstructed by other people, objects or overlying text, (3) video quality is sufficient for visual assessment of infant movements. All segments from these datasets were manually reviewed to confirm the absence of seizures while adhering to our inclusion criteria.

This study was approved by the Institutional Review Board of Charité—Universitätsmedizin Berlin (reference number EA2/273/23). For publicly available social media videos, we followed established ethical principles, including fair use doctrine for non-commercial research purposes, using only publicly accessible content, collecting no personally identifiable information beyond the videos themselves, and not publishing source links or screenshots to protect privacy. For the in-hospital video-EEG monitoring validation dataset, due to the retrospective nature of the analysis, informed consent of patients was waived by the ethics committee.

### Out-of-sample external testing

We collected three additional external datasets to further validate seizure detection performance and evaluate FAR: (1) smartphone videos of infants with ES published on YouTube after 2022 and on TikTok, collected using the same approach as for the derivation dataset. (2) An additional cohort of normally behaving infants from YouTube to assess FAR on smartphones. (3) Videos from long-term video-EEG monitoring recordings of infants under 2 years of age at the Epilepsy-Center Berlin-Brandenburg. For each video, we included all 5-s segments where the subject was clearly visible. Since in-hospital videos from long-term video-EEG monitoring were of long duration and captured with stationary cameras with a wide view, these videos were cropped automatically around the infant using an existing AI model designed for object detection^51^. For all in-hospital video segments used for FAR evaluation, seizure activity was ruled out by video-EEG.

### Meta-information and technical characteristics analysis

When available, we collected additional meta-information from the online videos of ES, including view count, likes, comments, and upload year, to assess the diversity and reach of this content. To evaluate the technical heterogeneity of the collected videos, we analyzed video resolution, duration, frame rate, and bitrate using the Python library OpenCV and FFmpeg. Additionally, we quantified general visual characteristics by analyzing brightness, daytime vs. nighttime recordings, motion, and sharpness of videos. Brightness was calculated as the mean pixel intensity across sampled frames, providing insight into lighting conditions. Nighttime recording was estimated using the averaged variance of the RGB pixels; if the average was below a predefined threshold, then the video was classified as recorded during night, otherwise during day. Motion was assessed by measuring the mean absolute difference between consecutive frames, indicating the level of movement or camera stability. Sharpness was evaluated using the variance of the Laplacian operator, offering a measure of image clarity.

### Video data preprocessing

To ensure consistency and to enhance the dataset for model training, we performed the following preprocessing steps: (1) we unified frame rates to 30 frames per second using FFmpeg. (2) We cut videos into 5-s segments, annotating each as seizure or non-seizure. (3) We applied several augmentations to each video segment, including vertical flip, horizontal flip, 90° clockwise and counterclockwise rotation, and color inversion to increase variability within our dataset. (4) We standardized frame size to 224 by 224 pixels by downsampling the resolution of each frame using a trilinear interpolation function. (5) We normalized color values per pixel using z-transformation to meet the requirements of the foundational model used^19^.

### Training and testing of a classification model

We trained our model using the Hiera Vision Transformer, which was pretrained on the Kinetics 400 Human Action Recognition dataset^18,19^. Deep learning for human action video analysis typically follows one of three main strategies: (1) extracting visual features from individual frames followed by recurrent neural network analysis; (2) extracting pose landmarks from frames followed by temporal analysis; or (3) using a foundational model pretrained on large video datasets to extract spatio-temporal features, which is then fine-tuned for specific tasks. In our pipeline, we selected the third approach as it has demonstrated superior performance in action recognition tasks with limited training data and may provide advantages in adaptability to variations in camera angles and lighting conditions. For feature extraction, our pipeline uses the Hiera Vision Transformer architecture, which directly learns relevant spatio-temporal features from the video data without requiring explicit motion quantification methods. The Hiera Vision Transformer was chosen for its hierarchical architecture that effectively captures both local and global spatio-temporal patterns, and its high performance on human action recognition benchmarks. For fine-tuning, we employed Low-Rank Adaptation (LoRA), a parameter-efficient fine-tuning technique that adapts pretrained weights while maintaining the model’s generalization capabilities^52^. Finally, the model was adapted for our purpose by modifying the final classification layer for binary classification using the sigmoid function, distinguishing between seizure and non-seizure video segments. We employed a five-fold cross-validation approach for training and testing on the derivation dataset. The data was split at the child level into 5 non-overlapping test folds, ensuring all segments from a single child were in the same fold. For hyperparameter optimization, we monitored learning curves and selected 0.0001 learning rate as optimal start point, multiplying by a factor of 0.75 every 10 epochs to ensure convergence and avoid overfitting. The batch size was set to 4 as a balance between computational efficiency and training stability. To improve specificity, additional data from 127 healthy infants were included in the training set only. Finally, we performed additional evaluations of model performance on three independent datasets, using a classification model trained on the entire derivation dataset.

### Performance metrics

We assessed the model’s performance using the area under the receiver-operating-characteristic curve (AUC), sensitivity, specificity, accuracy, and false alarm rate (FAR). For children who had only one class of video segments (i.e., either only seizure segments or only non-seizure video segments), AUC and the metric measuring the missed segment class were not calculated. We used the standard threshold of 0.5 to calculate sensitivity, specificity, and accuracy. We reported the FAR, evaluated on seizure-free children, as the percentage of normal segments incorrectly classified as seizures. We present all results using the mean and 95% confidence interval.

## Supplementary information

Supplementary Information

## Data Availability

Open-source smartphone video data is not shared publicly due to ethical reasons; however URLs are available upon reasonable request to qualified researchers. Video-EEG monitoring data is not publicly available due to patient privacy concerns.

## Code availability

Code for the foundational video model used is publicly available at https://github.com/facebookresearch/hiera. Code used for fine tuning is publicly available at https://github.com/microsoft/LoRA. The underlying code for training and testing is not publicly available for proprietary reasons. We used Python 3.9.13 with specific packages (torch 2.2.0, hiera-transformer 0.1.2) on an Intel(R) Xeon(R) Silver 4216 CPU with two NVIDIA RTX A6000 GPUs. Inference time was 0.73 s per each 5-s video segment.
