# Supplementary material for: Detection of epileptic spasms using foundational AI and smartphone videos
Source: NPJ Digit Med. 2025 Jun 17;8:370. doi: 10.1038/s41746-025-01773-1 (PMC12174323; doi:10.1038/s41746-025-01773-1)
Supplement: Supplementary file 1 — Supplementary Information [file 41746_2025_1773_MOESM1_ESM.pdf]

Supplementary Information

Supplementary Table 1. Epileptic spasm symptomatology across datasets

ID	**Dataset**	**Semiology**	**Involvement**	**Symmetry**	**Subtle Seizure**	**Seizure segment Count**
1	Derivation	Flexor	Arms, Face	Symmetric		10
2	Derivation	Mixed	Arms, Face	Asymmetric	Yes	10
2	Derivation	Flexor	All	Symmetric		12
3	Derivation	Extensor	Arms, Face	Symmetric		35
3	Derivation	Flexor	All	Symmetric		35
3	Derivation	Mixed	All	Symmetric		5
3	Derivation					0
4	Derivation	Flexor	All	Symmetric		5
4	Derivation	Flexor	All	Symmetric		4
5	Derivation	Flexor	Arms, Face	Symmetric	Yes	2
5	Derivation	Flexor	All	Symmetric		2
5	Derivation	Extensor	Arms, Legs, Face	Symmetric		3
6	Derivation	Mixed	All	Symmetric		2
6	Derivation	Flexor	All	Symmetric		9
7	Derivation	Flexor	Arms, Legs, Face	Symmetric	Yes	6
7	Derivation	Flexor	All	Symmetric		26
7	Derivation	Flexor	All	Symmetric		4
8	Derivation	Flexor	Arms, Face	Symmetric	Yes	30
8	Derivation	Flexor	All	Symmetric		5
9	Derivation	Flexor	Face, Neck, Trunk	Symmetric	Yes	4
9	Derivation	Flexor	Arms, Legs, Neck, Trunk	Symmetric		7
9	Derivation	Flexor	Arms, Face, Neck	Symmetric		1
10	Derivation	Flexor	All	Symmetric		8
10	Derivation	Flexor	All	Symmetric		12
10	Derivation	Flexor	All	Symmetric		8
11	Derivation					0
11	Derivation					0
12	Derivation	Extensor	Arms, Face	Symmetric	Yes	6
12	Derivation	Extensor	Arms, Face	Symmetric		3
13	Derivation	Mixed	All	Symmetric		4
13	Derivation	Flexor	Arms, Legs, Face, Neck	Symmetric		2
14	Derivation	Flexor	Arms, Face	Symmetric	Yes	12
14	Derivation	Extensor	Arms, Face	Symmetric		4
14	Derivation	Mixed	Arms, Legs, Neck, Trunk	Symmetric		3
14	Derivation					0
15	Derivation					0
15	Derivation					0

15	Derivation					0
15	Derivation					0
16	Derivation	Flexor	Face, Neck	Symmetric	Yes	3
16	Derivation	Flexor	Arms, Face, Neck, Trunk	Symmetric		2
17	Derivation	Flexor	All	Symmetric		2
18	Derivation	Flexor	Arms, Legs, Face	Asymmetric		13
19	Derivation					0
20	Derivation	Mixed	Arms, Legs	Symmetric		13
21	Derivation	Flexor	Arms, Legs, Face, Neck	Symmetric		2
22	Derivation					0
23	Derivation	Extensor	Arms, Face	Asymmetric	Yes	2
24	Derivation	Flexor	All	Symmetric		1
25	Derivation	Extensor	Face, Arms	Asymmetric	Yes	3
26	Derivation					0
27	Derivation					0
28	Derivation	Flexor	Arms, Legs	Symmetric		2
29	Derivation	Flexor	All	Symmetric		5
30	Derivation	Flexor	All	Symmetric		2
31	Derivation	Flexor	Arms	Asymmetric	Yes	12
32	Derivation	Flexor	Arms, Legs	Symmetric	Yes	4
33	Derivation	Flexor	Arms, Legs, Face	Symmetric		2
34	Derivation	Mixed	Arms, Legs, Face	Asymmetric	Yes	5
35	Derivation	Mixed	Arms, Face	Symmetric		5
36	Derivation	Flexor	All	Symmetric		4
37	Derivation	Extensor	Arms, Legs, Neck	Asymmetric		3
38	Derivation	Extensor	Arms, Legs, Face	Symmetric		3
39	Derivation	Extensor	All	Symmetric		2
40	Derivation	Extensor	Arm, Face	Asymmetric	Yes	4
41	Derivation	Extensor	Arms, Legs, Face	Symmetric		4
42	Derivation	Extensor	Arms, Legs, Face	Symmetric		3
43	Derivation	Flexor	Arms, Neck, Face, Trunk	Symmetric		4
44	Derivation	Mixed	All	Symmetric		16
45	Derivation	Extensor	Arms, Legs, Face	Symmetric		8
46	Derivation	Extensor	Arms, Face	Symmetric		7
47	Derivation	Mixed	All	Symmetric		4
48	Derivation	Flexor	Arms, Face, Neck, Trunk	Symmetric		5
49	Derivation	Flexor	Arms, Face	Asymmetric		4
50	Derivation	Flexor	Arms, Face	Symmetric	Yes	3
51	Derivation	Mixed	All	Symmetric		4
52	Derivation	Flexor	Arms, Face, Neck, Trunk	Symmetric		5
53	Derivation	Flexor	Arms, Face, Neck	Symmetric		3
54	Derivation	Mixed	Arms, Face	Asymmetric	Yes	4

55	Derivation	Extensor	Arms, Face	Symmetric		2
56	Derivation	Extensor	Arms, Face		Yes	14
57	Derivation	Flexor	Face, Neck, Trunk	Symmetric		8
58	Derivation	Flexor	Face, Neck		Yes	6
59	Derivation	Flexor	Face, Neck	Symmetric		17
60	Derivation	Mixed	All	Symmetric		18
61	Derivation	Flexor	Arms, Face, Neck, Trunk	Symmetric		23
62	Derivation	Flexor	Face, Arms	Symmetric	Yes	2
63	Derivation	Flexor	Arms, Legs, Neck	Symmetric		13
64	Derivation	Flexor	Arms, Neck, Face, Trunk	Symmetric		3
65	Derivation	Flexor	Arms, Neck, Face, Trunk	Symmetric		3
66	Derivation	Flexor	Arms, Legs	Symmetric		8
67	Derivation	Flexor	Arms, Neck, Face, Trunk	Symmetric		3
68	Derivation	Mixed	Arms, Legs, Neck	Symmetric		7
69	Derivation	Flexor	Arms, Face, Neck	Symmetric		4
70	Derivation	Flexor	Arms, Legs, Neck, Trunk	Symmetric		8
71	Derivation	Extensor	Arms	Symmetric		3
72	Derivation	Flexor	Arms, Legs, Face	Symmetric	Yes	22
73	Derivation					0
74	Derivation	Mixed	All	Symmetric		5
75	Derivation	Flexor	Arms, Legs, Face	Symmetric		1
76	Derivation	Mixed	Arms, Legs, Face	Symmetric		17
77	Derivation	Flexor	Arms, Face, Neck, Trunk	Symmetric		6
78	Derivation					0
79	Derivation					0
80	Derivation					0
81	Derivation	Flexor	Arms, Neck, Face	Symmetric		16
82	Derivation	Mixed	Arms, Neck, Face	Symmetric		7
83	Derivation	Flexor	Arms, Legs, Neck, Face	Asymmetric		7
84	Derivation	Flexor	All	Symmetric		7
85	Derivation	Flexor	All	Symmetric		2
86	Derivation	Flexor	Arms, Face, Neck	Symmetric		6
87	Derivation	Flexor	Arms, Legs, Face	Symmetric		2
88	Derivation	Flexor	Arms, Face	Asymmetric		9
89	Derivation					0
90	Derivation	Flexor	Arms, Legs	Symmetric		3
91	Derivation	Flexor	All	Symmetric		11
92	Derivation	Flexor	All	Symmetric		5
93	Derivation					0
94	Derivation	Mixed	Arms, Face, Neck	Symmetric		2

95	Derivation	Flexor	Arms, Legs	Symmetric		1
96	Derivation	Flexor	Arms, Legs, Face	Symmetric		6
97	Derivation	Extensor	Arm, Neck	Asymmetric	Yes	10
98	Derivation	Flexor	Face, Legs		Yes	5
99	Derivation	Mixed	All	Symmetric		14
100	Derivation	Flexor	All	Symmetric		6
101	Derivation	Mixed	Arms, Face, Neck, Trunk	Symmetric		2
102	Derivation	Extensor	Arms, Face	Symmetric	Yes	6
103	Derivation	Extensor	Arms, Legs, Face	Symmetric		8
104	Derivation	Flexor	Arms, Neck	Symmetric		6
105	Derivation	Mixed	Arms, Legs, Trunk	Symmetric		8
106	Derivation	Flexor	All	Symmetric		8
107	Derivation	Flexor	Arms, Face, Neck	Asymmetric		2
108	Derivation	Flexor	Arms, Face, Neck	Symmetric		1
109	Derivation	Extensor	Arms, Face	Symmetric		1
110	Derivation	Extensor	Arms, Face	Symmetric		4
111	Derivation	Flexor	All	Symmetric		16
112	Derivation	Flexor	All	Symmetric		2
113	Derivation	Flexor	Arms, Face, Neck	Symmetric		2
114	Derivation	Flexor	Arms, Legs, Face, Neck	Symmetric		4
115	Derivation	Flexor	Arms, Face	Asymmetric	Yes	5
116	Derivation	Flexor	All	Symmetric		5
117	Derivation	Mixed	Arms, Legs	Symmetric	Yes	15
118	Derivation	Mixed	Arms, Legs	Symmetric		3
119	Derivation	Flexor	Arms, Legs	Symmetric		3
120	Derivation	Flexor	Arms, Face	Symmetric		3
121	Derivation	Extensor	Arms, Legs	Symmetric		22
122	Derivation	Mixed	All	Symmetric		3
123	Derivation	Extensor	Arms, Face	Symmetric	Yes	12
124	Derivation	Mixed	All	Symmetric		8
125	Derivation	Flexor	All	Symmetric		21
126	Derivation	Flexor	Arms, Face, Neck	Asymmetric	Yes	3
127	Derivation	Mixed	Arms, Legs, Face	Symmetric		14
128	Derivation	Mixed	Arms, Legs, Face	Symmetric		3
129	Derivation	Flexor	Face, Neck	Symmetric	Yes	3
130	Derivation	Flexor	All	Symmetric		3
131	Derivation	Flexor	Arms, Legs, Neck	Asymmetric		4
132	Derivation	Mixed	All	Symmetric		3
133	Derivation	Extensor	Arms, Face	Symmetric		11
134	Derivation	Flexor	Arms, Face	Symmetric		1
135	Derivation	Extensor	Arms, Face	Symmetric	Yes	3
136	Derivation					0
137	Derivation	Flexor	Arms, Face, Neck, Trunk	Symmetric		5
138	Derivation	Flexor	All	Symmetric		2

139	Derivation	Flexor	All	Symmetric		2
140	Derivation	Flexor	All	Symmetric		2
141	Derivation	Flexor	Arms, Face	Symmetric	Yes	3
142	External Validation 1	Flexor	All	Symmetric		9
143	External Validation 1					0
144	External Validation 1	Flexor	Arms, Legs, Face	Symmetric		3
145	External Validation 1	Flexor	Arms, Legs, Face, Neck	Symmetric		3
146	External Validation 1	Mixed	Arms, Legs, Face	Symmetric		3
147	External Validation 1	Flexor	Arms, Legs, Face	Symmetric		6
148	External Validation 1	Flexor	Arms, Legs, Face, Neck	Symmetric		1
149	External Validation 1	Extensor	Arms, Face	Symmetric	Yes	4
150	External Validation 1	Flexor	Arms, Legs	Symmetric		3
151	External Validation 1					0
152	External Validation 1					0
153	External Validation 1	Flexor	Arms, Face, Neck, Trunk	Asymmetric		2
154	External Validation 1	Flexor	Arms, Face, Neck	Symmetric		3
155	External Validation 1	Flexor	All	Symmetric		3
156	External Validation 1	Flexor	Arms, Legs, Face, Neck	Symmetric		5
157	External Validation 1					0
158	External Validation 1	Flexor	Arms, Face	Symmetric		2
159	External Validation 1	Extensor	Arms	Symmetric	Yes	5
160	External Validation 1	Flexor	All	Symmetric		2
161	External Validation 1					0
162	External Validation 1	Flexor	All	Symmetric		2
163	External Validation 1	Flexor	All	Symmetric		4

164	External Validation 1	Flexor	Arms, Face	Symmetric	Yes	2
165	External Validation 1	Flexor	Arms, Legs, Face, Neck	Symmetric		4
166	External Validation 1	Extensor	Arms, Legs, Face	Symmetric		1
167	External Validation 1	Flexor	All	Symmetric		3
168	External Validation 3	Extensor	Arms, Legs, Face, Neck	Symmetric		45

Table contains the description of all semiological characteristics of all the subjects in the derivation dataset, external validation datasets 1 and 3 . Multiple rows with the same subject ID denote multiple semiologies from different videos.

Supplementary Table 2. Technical characteristics of videos by model performance

Technical Characteristics	Derivation dataset		Dataset 1 - Smartphone - Epileptic Spasm Cohort		Dataset 2 - Smartphone - Normally Behaving Infants		Dataset 3 – Hospital- Video-EEG Monitoring	
	True Predictions	False predictions	True Predictions	False predictions	True Predictions	False predictions	True Predictions	False predictions
Resolution, high/medium/low %	54/4/42	61/2/37	89/8/3	100/0/0	69/25/7	80/20/0	2/77/21 *	3/70/27 *
Bitrate, kbps median	528.0	584.5	1125.0	1074.0	2165.0	1410.0	3545.0	3496.0
Brightness, median gray level intensity	104.1	107.2	117.0	127.8	124.8	115.8	107.9	118.0
Sharpness values, Laplacian variance	90.7	80.9	313.3	292.2	60.2	86.3	285.7**	207.6**
Motion median frame absolute difference	192.7	197.4	202.2	224.1	185.6	208.8	89.1**	73.4**

True predictions include true positives and true negatives. False predictions include false positives and false negatives. High resolution is defined as above 720p, medium resolution between 480-720p, and low resolution under 480p. Abbreviations: kbps - kilobytes per second. * p-value < 0.05, ** p-value < 0.01, mann whitney U test

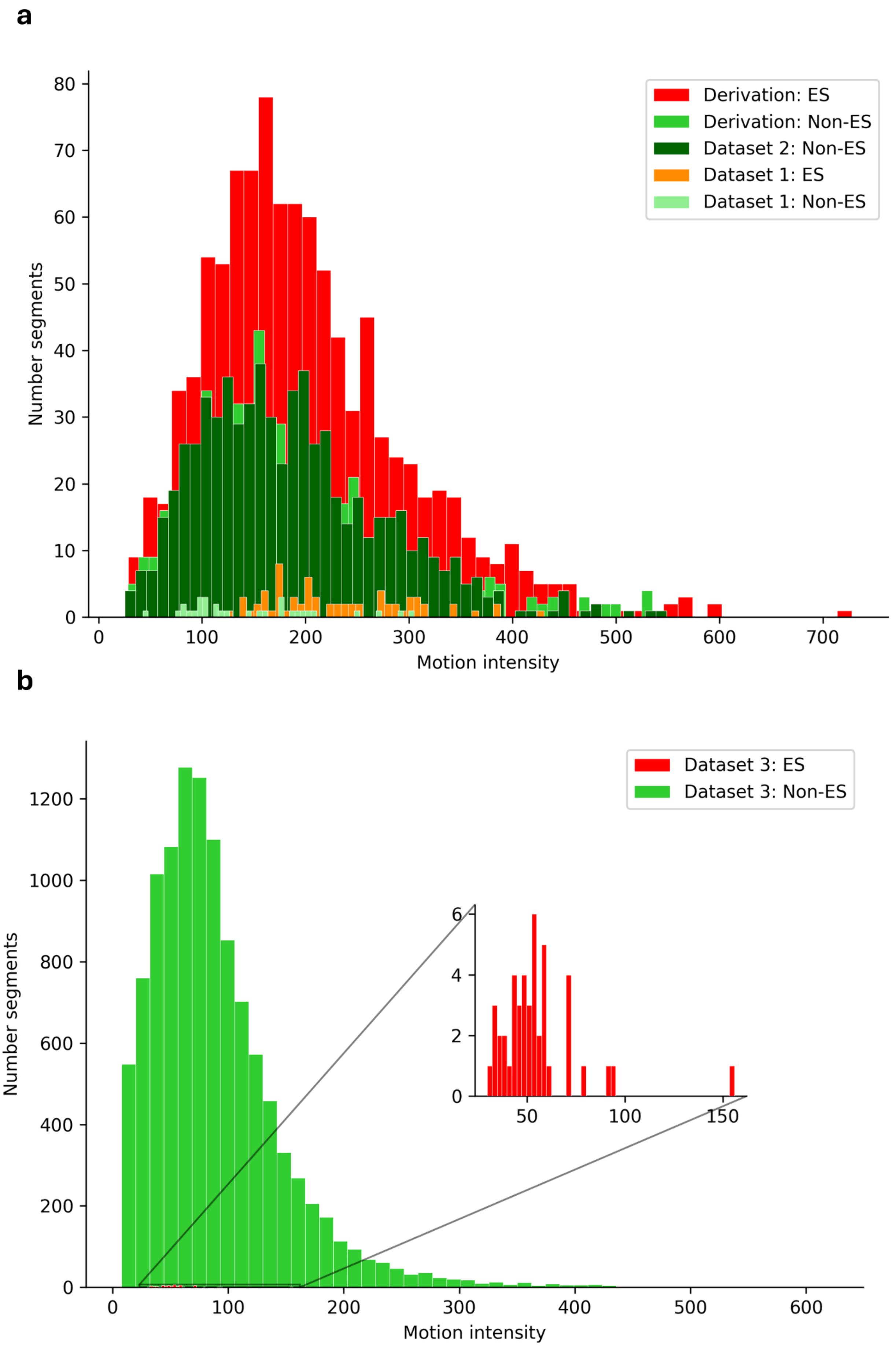
a
Derivation: ES
Derivation: Non-ES
Dataset 2: Non-ES
Dataset 1: ES
Dataset 1: Non-ES
Number segments
0
10
20
30
40
50
60
70
80
0
100
200
300
400
500
600
700
Motion intensity
b
Dataset 3: ES
Dataset 3: Non-ES
Number segments
0
200
400
600
800
1000
1200
0
100
200
300
400
500
600
Motion intensity
0
2
4
6
50
100
150

Supplementary Figure 1: Movement intensities across datasets and classes. Figure shows distribution of movement intensities of all included video segments. **a** includes all video segments in the smartphone based datasets (derivation dataset, external validation datasets 1+2). **b** shows all video segments in the hospital based external validation dataset 3. Colors represent datasets and classes. The x-axes show motion intensity calculated as the mean absolute difference between consecutive frames. The y-axes show number of segments. These plots demonstrate comparable distribution ranges of movement intensities across smartphone datasets and classes (ES vs. non-ES video segments). Comparing performance accuracy in video segments within the lowest movement quantile (Q1=0-25%) and the highest movement quantile (Q4=75-100%) we found no significant difference In the derivation dataset (accuracy Q1=84% vs. Q4=83%, $p=0.77$, Mann-Whitney U test), external dataset 1 (accuracy Q1=96% vs. Q4=88%, $p=0.31$), and external dataset 2 (accuracy Q1=99% vs. Q4=99%, $p=1.0$). In dataset 3 (accuracy Q1=93% vs. Q4=97%, $p<0.001$) we found better performance with higher intensity movements.
